# Simulation-based skills training in non-performing orthopedic surgeons: skills acquisition, motivation, and flow during the COVID-19 pandemic

**DOI:** 10.1080/17453674.2020.1781413

**Published:** 2020-06-23

**Authors:** Leif Rune Hedman, Li Felländer-Tsai

**Affiliations:** a Department of Clinical Science, Intervention and Technology (CLINTEC), Division of Orthopedics and Biotechnology, Karolinska Institutet, Stockholm, Sweden, and Center for Advanced Medical Simulation and Training (CAMST), Karolinska University Hospital, Stockholm;; b Department of Psychology, Umeå University, Umeå, Sweden

The COVID-19 pandemic will continue to have a profound effect on society, including orthopedic surgery (Felländer-Tsai 2020, Wright et al. 2020). Educational crisis management is mandated. As elective surgeries are being postponed for many orthopedic residents they must change their daily clinical routines. Increased opportunities for virtual and simulated surgical training can facilitate trainees/residents/experts retaining basic competence. A way to maintain skills acquisition and to prevent skill decay is to provide increased possibilities to practice simulated surgical tasks, i.e., psychomotor training including virtual learning such as 3D visualization.

For a growing number of minimally invasive and technically challenging orthopedic procedures, there is a movement to improve surgical skills training outside the operating room because of, e.g., patient safety concerns (Atesok et al. 2016). Surgical simulation is a powerful tool that can help meet these training demands (Felländer-Tsai et al. 2004, Gallagher et al. 2005, Johnston et al. 2016). Kogan et al. (2020) pointed out that quarantine away from patient care, active social distancing, and diminished case volume during the pandemic will increase the need for surgical simulation training.

## ^Motivation in simulation training^

Simulation-based skills training including 3D visualizations is becoming increasingly integrated into surgical education as an important teaching method across most surgical specialties. Proficiency and progression-based simulation training is today an influential approach to surgical simulation-based skills training and medical education (Gallagher et al. 2005, Ahlberg et al. 2007, Stefanidis et al. 2010). This kind of training is considered to improve general motivation, skills training, and skill retention.

Wallace et al. (2017) have remarked that most of the studies in the field of cognitive training are without underlying theory. The psychological mechanisms underlying the efficacy of cognitive training programs are not well known. There is also a lack of theoretically based studies of motivational processes in surgical simulation training.

Self-determination theory (SDT) claims that when the basic needs for competence, autonomy, and relatedness are satisfied, personal well-being and social development are optimized (Deci and Ryan 2000). In this condition individuals are intrinsically motivated, able to fulfill their potential, and able to seek out progressively greater challenges. The intrinsic types of motivation are the most self-determined and are performed for the satisfaction gained from the activity. Extrinsic motivation lies at the lower end of the self-determination scale. The extrinsic motivation types ranging from most self-determined to least self-determined are: identified regulation; introjected regulation; and external regulation. Amotivation is characterized by the absence of motivation. The different types of motivation postulated by SDT have proved to be meaningful in order to predict the level of engagement in a variety of life domains. Research has shown that there is a strong positive correlation between intrinsic motivation and good work performance (Deci and Ryan op.cit).

Csikszentmihályi et al. (2005) defined flow as a mental experience of intensive enjoyment characterized by complete concentration, heightened sense of control, merging of action and attention, loss of self-consciousness, distortion of time perception, and internally driven. Flow may be described as a temporary psychological state of arousal/attention involving positive feelings during a specific moment-to-moment activity. Flow treated as a state, assessed by its intensity, can foster positive affect and stimulate positive educational outcomes (Cerasoli et al. 2014, Csikszentmihályi 2014). According to flow theory, flow can increase intrinsic motivation toward a specific activity (Moneta 2012, De Fraga and Moneta 2016).

Flow experience has been studied in fields such as performing arts and sport, education, neuropsychology, educational psychology, social psychology work, and everyday activities (De Fraga and Moneta op.cit).

The extensively validated simulator for generic minimally invasive navigational skills, MIST VR (Gallagher et al. 2005), and also newer and more advanced simulators, have long proved to be very suitable training environments. We have identified state motivation as an important variable for minimally invasive surgical performance both when training in surgical simulators and during acute team training (Schlickum et al. 2013). Moreover, a state of flow appears to contribute to both real-life and simulated learning of minimal invasive skills (Ahlborg et al. 2015).

## ^Engagement in a simulated basic skill task: example from a pilot experiment^

There is still a lack of detailed knowledge of different motivational states while reaching a predetermined criterion level of performance. As part of the previously published investigation (Schlickum et al. 2016) the present authors collected and analyzed data on flow experience and situational motivation from 30 surgical novices. All received a standardized verbal introduction and a standardized video-demonstration of a well-studied basic task (manipulative diathermy at medium difficulty) in a validated minimally invasive surgery training-virtual reality simulator (MIST-VR). Training was then performed for approximately 1 hour until a pre-set criterion level was reached following measurement of performance. Motivation was then self-assessed by validated instruments: a short and long flow state scale (Jackson et al. 2008), and a situational motivation scale (Guay et al. 2000). We found no statistically significant correlations between types of situational motivation and MIST-VR performance. Presumably, the manipulation task in this low-fidelity simulator was too simple. However, flow state scores for the short and long version of the flow scales were positively correlated, and flow state assessed by the short flow state scale was correlated with the best performance (data not shown). Notably, flow state assessed by the short state flow scale were positively correlated with intrinsic motivation (data not shown). The pilot experiment indicates that surgical novices’ flow state was correlated to performance in the simulated basic skills task.

## ^The future^

Findings in training research should be considered when new training programs have to be developed, delivered, and monitored. Salas et al. (2012) have provided evidence-based recommendations for maximizing training effectiveness before, during, and after training. We recommend using their checklists. They provide insight into best practices for training skills that are durable and resistant to skill decay, assessing skills over time, and repeating training at appropriate intervals. Most of the proposed actions will promote motivation to learn and increase flow. Moreover, to foster motivation during skill acquisition in simulation training, feedback of performance is critical and given: (1) automatically and immediately by the simulator itself, (2) by the instructor giving verbal feedback during training (technical and pedagogical support), and (3) by the instructor and other team members after the training sessions (debriefing). All kinds of feedback can offer opportunities to tailor the trainee’s abilities and feelings during the process of skill acquisition. As in ideomotoric training (Immenroth et al. 2007), where the surgeon imagines him- or herself performing a procedure, the sensory aspects of the procedure should be included as far as possible in order to elicit motivational and arousal components of cognitive training.

Atesok et al. (2016) have reviewed several barriers to overcome when integrating the simulation of surgical skill training into orthopedic surgical education. They discuss the value of proficiency and progression-based simulator training as the next-generation approach, and using measurable criteria for technical performance. The outcome measures of technical skill performance should be quantitative metric. The model presented by Khamis et al. (2016) integrates principles of curriculum development and simulation design. It involves best practices for, e.g., individualized learning, providing formative and summative feedback, deliberate practice with formative feedback, and mastery learning progressing from novice, competent, proficient, expert to master.

## ^Conclusion^

When used appropriately, extended simulation training can be a highly effective additional training tool in the development and maintenance of technical skills and combatting skills decay, taking into account motivation and flow. This is relevant also for temporarily non-performing orthopedic surgeons during a crisis affecting the organization of healthcare such as the COVID-19 pandemic.
